# The Time Course of Spatial Attention Shifts in Elementary Arithmetic

**DOI:** 10.1038/s41598-017-01037-3

**Published:** 2017-04-19

**Authors:** Dixiu Liu, Danni Cai, Tom Verguts, Qi Chen

**Affiliations:** 1 School of Psychology, South China Normal University, 510631 Guangzhou, China; 2 Center for Studies of Psychological Application, South China Normal University, 510631 Guangzhou, China; 3 Guangdong Key Laboratory of Mental Health and Cognitive Science, South China Normal University, 510631 Guangzhou, China; 4 Department of Experimental Psychology, Ghent University, 9000 Ghent, Belgium

## Abstract

It has been proposed that elementary arithmetic induces spatial shifts of attention. However, the timing of this arithmetic-space association remains unknown. Here we investigate this issue with a target detection paradigm. Detecting targets in the right visual field was faster than in the left visual field when preceded by an addition operation, while detecting targets in the left visual field was faster than in the right visual field when preceded by a subtraction operation. The arithmetic-space association was found both at the end of the arithmetic operation and during calculation. In contrast, the processing of operators themselves did not induce spatial biases. Our results suggest that the arithmetic-space association resides in the mental arithmetic operation rather than in the individual numbers or the operators. Moreover, the temporal course of this effect was different in addition and subtraction.

## Introduction

Several studies have demonstrated that there is a strong link between number and space^1–5^. Recently, the investigation of this number-space association has been extended to mental arithmetic^6–9^. In a seminal study, McCrink *et al*.^9^ observed a systematic bias toward larger values for addition problems and toward smaller values for subtraction problems in non-symbolic arithmetic (operational momentum effect). Further empirical evidence for an arithmetic-space relation came from Knops *et al*. who found that an arithmetic operation spatially biased subsequent responses^8^. Specifically, subjects tended to select options on the right side for addition problems, and options on the left side for subtraction problems^8^. The spatial biases during addition and subtraction were reflected in activation patterns in parietal cortex, which resemble the activation pattern produced by rightward or leftward eye movement respectively^7^. It was hypothesized that addition (subtraction) elicits covert movements to the right (left) along the mental number line^8–14^. According to this account, spatial shifts in mental arithmetic rely on the spatial nature of number representations, and are induced by number processing. A different but related explanation is the neuro-computational model of Chen & Verguts^6^. This model proposed that numbers are not inherently spatial, but are transformed into spatial representations for the purpose of mental arithmetic^15^. After solving the arithmetic problem in a spatial coordinate frame, the resulting spatial coordinate is then transformed back into the number domain, yielding a specific (and hopefully correct) numerical answer.

Recent studies started to investigate the locus and timing of spatial shifts of attention in mental arithmetic^16–18^. Masson and Pesenti^17^ found that 450 ms after solving single-digit subtraction problems, the detection of targets on the left side was facilitated. No such acceleration was found for right side targets after solving single-digit addition problems. For two-digit arithmetic problems, visuospatial attention shifts were found in addition instead of subtraction. At an earlier stage (onset of the second operand), Mathieu *et al*.^18^ found that addition problems were solved faster when the second operand was presented to the right than to the left, whereas subtraction problems were solved faster when the second operand was presented to the left than to the right. The time window in which the arithmetic-space association occurred was different for the two operations. It occurred earlier in addition (150 ms after operator presentation) than in subtraction (300 ms after operator presentation).

It has also been suggested there is a relation between the processing of the operator itself and space^19–21^. However, the nature of such an operator-space relationship remains unclear. For example, Pinhas *et al*.^21^ used an operation sign classification task, and found that left side responses were faster for the minus sign than for the plus sign, whereas right side responses were faster for the plus sign than for the minus sign. However, this interaction might be due to semantic associations, such as “left-minus” or “right-plus”^11, 22^. Further, Hartmann *et al*.^19^ confirmed an operator-space association by analyzing spontaneous eye movements during mental arithmetic. The gaze position was located more upward for “plus” when compared to “minus”, and this significant difference in vertical space was first detected after the onset of the operator. However, in Hartmann *et al*.’s study the operator was always presented 760 ms after the first operand. Therefore, it is possible that the operator-space interaction was at least partially due to this operand.

Thus, despite this recent interest, important questions remain unanswered. Firstly, it is unclear whether this arithmetic-space association originates from the mental calculation process (operation-space association); or instead originates from its constituents, for example, an association between number and space or between an operator and space. To address this, it is necessary to deconfound the operation-space association from number-space and operator-space interactions. Secondly, if mental arithmetic operation can induce spatial shifts of attention, when (at what stage) do they occur? Although Masson *et al*.^17^ found the arithmetic-space association at 450 ms after mental calculation, the single stimulus onset asynchrony (SOA) of 450 ms cannot uncover the time course of attention shifts. Indeed, the effect may also arise at earlier stages of mental arithmetic^6^, such as at the onset of the second operand.

**Figure 1:**
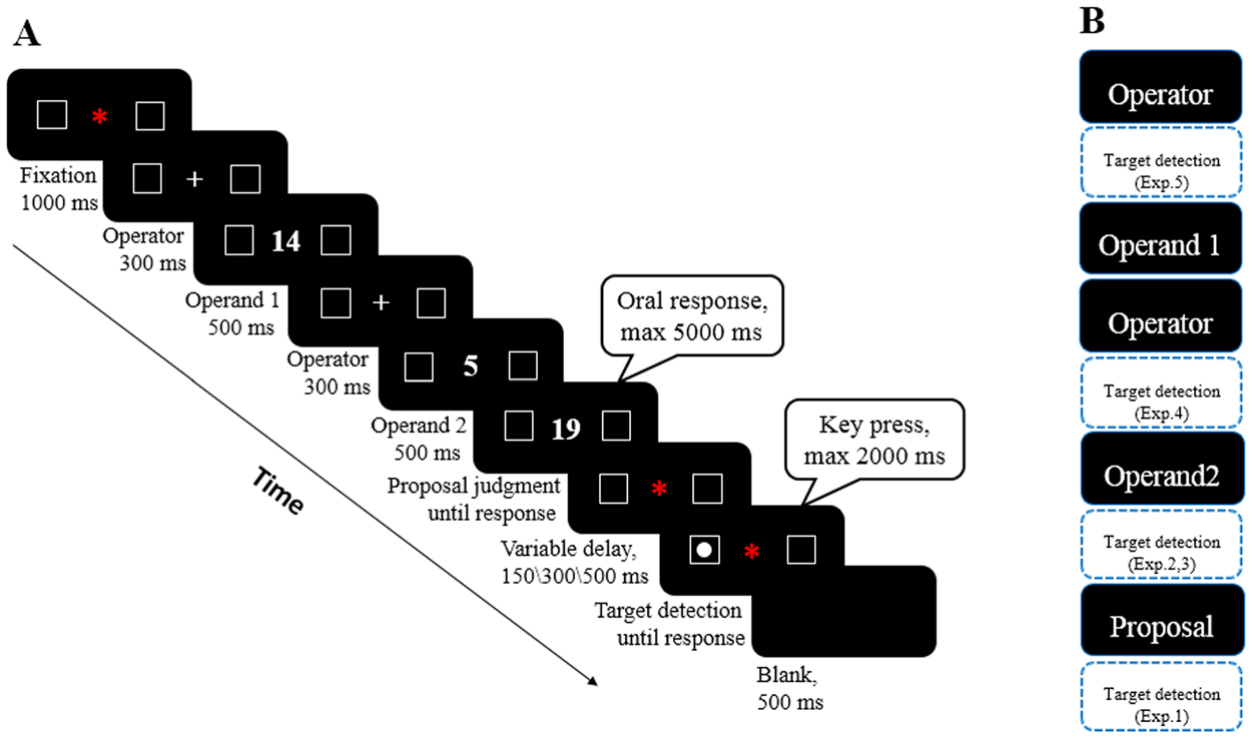
(**A**) Task sequence and timing of a sample trial. The operator, first operand, second operand and the proposed result (proposal) were presented sequentially at the center of the screen. The target (a white solid circle) was randomly presented on either the left or right side on 80% of all trials. Targets disappear as soon as participants respond, or remain on the screen with a maximum duration of 2000 ms. Similarly, the proposal would not disappear until participants give an oral response through a headset microphone, but with a maximum duration of 5000 ms. (**B**) General trial structure across the five experiments. Blue rectangles with dotted line indicate the location of the target detection task, which is different from Experiment 1 to 5. Stimuli for the arithmetic task (same in each experiment) are depicted by black filled boxes.

In the present study, we used a target detection task in combination with an arithmetic task to address these issues. Specifically, on each trial participants performed two tasks: (1) solving an arithmetic problem and judging whether the proposed result (proposal) was correct or incorrect (mental arithmetic task), (2) detecting whether the target (a white solid circle) was present or not (target detection task) (see Fig. 1A). The latter task measured where attention was located in space (left or right). The core sequence and timing of a trial was similar to the detection experiment in Fischer *et al*.^10^. In the following five experiments, we systematically matched the magnitude of operands or proposal in addition and subtraction to disentangle number-space from arithmetic-space interactions. To investigate the time course of arithmetic-space association, the target detection task was located at different stages of the arithmetic operation for different experiments (see Fig. 1B). In Experiment 1, the target detection task appeared after the proposal. In subsequent experiments, it appeared successively earlier: after the second operand (Experiment 2 and 3) or before the second operand (Experiment 4). In Experiment 5, the target detection task was implemented before the onset of the first operand. Furthermore, in all five experiments we adopted three variable delay times (150, 300 and 500 ms) before target detection to capture any potential spatial shifts of attention^10^.

## Experiment 1

### Probing after the proposal, and matching proposals in addition and subtraction

In Experiment 1, we intended to replicate whether solving arithmetic problems could induce spatial shifts of attention with target stimuli closely following the proposal. Proposal magnitudes were matched for addition and subtraction to eliminate confounds between number-space and arithmetic-space associations.

### Method

#### Participants

27 undergraduates (10 males, 25 right-handed) took part in Experiment 1. One of them was excluded from further analysis because his accuracy was lower than 80%. The remaining 26 subjects ranged in age from 19 to 24. All participants had normal or corrected-to-normal vision, and they were naïve with respect to the objective of the study.

The present study was approved by the Human Research Ethics Committee for Non-Clinical Faculties, School of Psychology, South China Normal University. All experimental procedures and other relevant details were carried out in accordance with the approved guidelines as well as the ethical guidelines. We obtained informed consent from all subjects before the experiment.

#### Stimuli

The main arithmetic problems were constructed with the criteria used by Knops and Viarouge^8^. In Experiment 1 (see Supplementary Materials Table S1), the first operand was 14, 28 or 56 for addition and 32, 64, or 128 for subtraction. The second operand was created in relation to the first operand, with a fixed proportion of the first operand: 28%, 49%, and 76% in addition; and 23%, 34%, and 44% in subtraction. Thus, the combinations of these three initial values (i.e., first operands) and their corresponding change (i.e., second operands) generated a 3 × 3 = 9 stimulus set for addition and subtraction. The proposals consisted of the correct answer and four deviant answers. These deviant answers were generated as round (c × 2^i/4^), where c was the correct answer and i ranged from −2 to 2. On 50% of the trials, the proposal was correct. The deviant answers were allocated in a counterbalanced way in each block.

#### Task and procedure

All experiments were conducted on an IBM PC equipped with a 17-inch screen. Stimulus presentation and data collection were programmed using E-Prime 2.0 software. An example trial can be seen in Fig. 1A. First, a red fixation “*” appeared in the center of the screen for 1000 ms together with two lateral boxes. One was left of the fixation cross and the other was right. As soon as the fixation disappeared, an operator (+ or −) replaced the fixation for 300 ms indicating the subsequent operation to be performed. Then, the first operand (O1) (500 ms), operator (300 ms), and the second operand (O_2_) (500 ms) were presented successively. After O_2_ was removed, a proposal was presented. Participants were instructed to make an oral judgment as soon and as accurately as possible on whether the proposal was correct or incorrect (“Dui (Yes)” or “Cuo (No)”). The proposal remained on the center of the screen until response. As soon as the proposal disappeared, and after a random delay (150, 300 or 500 ms), a target (a white solid circle) randomly appeared inside either the left or the right box on 80% of all trials. To prevent anticipatory responses, the other 20% of the trials were catch trials where no target appeared. Observers were asked to press the space bar with their preferred hand as quickly as possible when they had detected the target. The target remained on screen until response or until 2000 ms were passed.

Before testing, participants were instructed that arithmetic operations were irrelevant to target detection. They were also required to keep their eyes fixated on the center of the screen and not to make any attention shifts during the task. The task consisted of 360 experimental trials, which were administered in four consecutive blocks. After each block, the subjects were given a chance to rest. There was a training session consisting of 16 practice trials before the first experimental block.

### Results and Discussion

Trials with error responses (to either arithmetic or target detection task) were excluded from further analysis (9.3%). Moreover, the following trials were also excluded: (1) in the arithmetic task, trials where the microphone failed to trigger, or the judgment RT was more than 5000 ms (1.5%), (2) in the target detection task, trials where the target detection time was smaller or larger than three standard deviations from the mean for each participant (1.8%). The same exclusion criteria were applied in all experiments. Here and elsewhere, we only report the target detection task data. Mean RT (and SD) of the arithmetic task as a function of Operation, Target side, and Delay in all experiments appear in Supplementary Materials Table S4.

**Figure 2:**
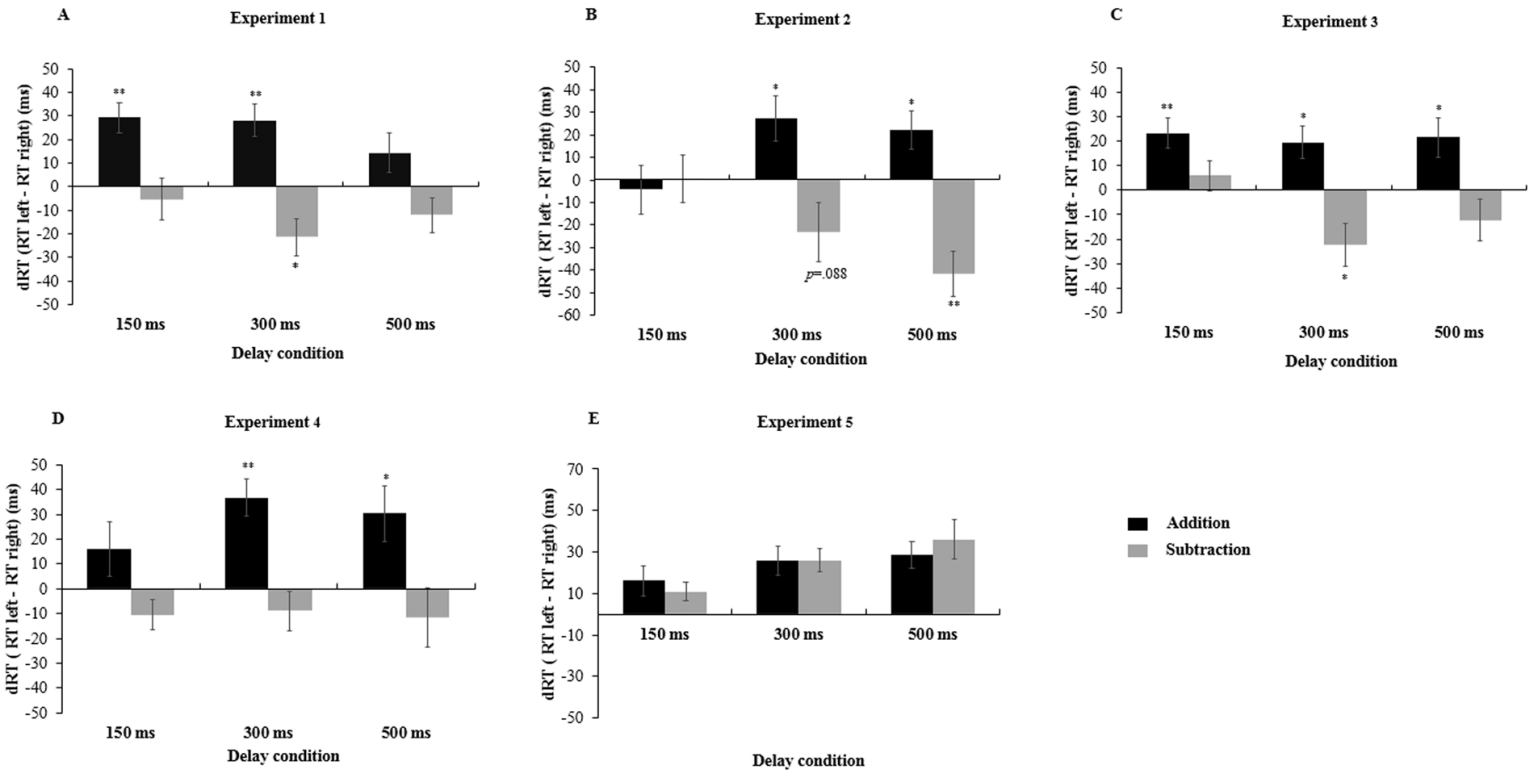
The difference in RT (dRT) as a function of operation (addition or subtraction) with 150 ms, 300 ms, and 500 ms delay in the five experiments. dRT, the mean RT of trials in which target appeared on the left, subtracted from the mean RT of trials in which target appeared on the right, is shown on the vertical axis. “Up” means “right faster”, while “down” means “left faster”. Error bars represent standard error of the mean (SEM). *p < 0.05, **p < 0.01. Here and elsewhere, data for the target detection task only are shown (see main text).

A 2 × 2 × 3 repeated measures analysis of variance (ANOVA) was carried out on mean RTs of the target detection data with operation (addition, subtraction), target side (left, right) and delay (150, 300 and 500 ms) as within-subject factors. See Table 1 for a full list of mean RT (and SD) and Fig. 2A for mean effects. There was a main effect of delay, *F* (2, 50) = 11.058, *p* < 0.05, *η* ^2^ = 0.307. Mean RTs were fastest in the 300 ms condition, which is significantly faster than the 150 ms condition, *F* (1, 25) = 5.968, *p* < 0.05, but not different from the 500 ms, *F* (1, 25) = 2.958, *p* > 0.05. Crucially, there was a significant interaction between operation and target side, *F* (1, 25) = 28.959, *p* < 0.05, *η* ^2^ = 0.537. The simple effect analysis showed that right-side targets were detected faster than left-side targets when it was preceded by an addition operation, *F* (1, 25) = 21.126, *p* < 0.05, *η* ^2^ = 0.458. When solving a subtraction problem, participants were faster at detecting left-side targets than right-side targets, *F* (1, 25) = 5.850, *p* < 0.05, *η* ^2^ = 0.190. Other main effects and interaction were not found.

**Table 1:** Mean RT (and SD) of the target detection task as a function of Operation, Target side, and Delay (in ms) in five experiments.

	Addition			Subtraction		
	150	300	500	150	300	500
Experiment 1						
Left	451 (67)	429 (83)	430 (59)	450 (71)	404 (71)	421 (52)
Right	422 (72)	401 (65)	416 (64)	456 (84)	425 (60)	433 (61)
Experiment 2						
Left	516 (100)	513 (117)	519 (95)	544 (102)	520 (106)	487 (86)
Right	520 (87)	485 (90)	497 (94)	544 (95)	543 (113)	529 (101)
Experiment 3						
Left	489 (73)	464 (75)	470 (80)	498 (74)	453 (76)	466 (75)
Right	465 (67)	444 (68)	449 (73)	492 (71)	475 (79)	478 (76)
Experiment 4						
Left	566 (137)	560 (144)	556 (130)	550 (134)	646 (133)	542 (136)
Right	547 (128)	521 (124)	522 (121)	565 (150)	555 (137)	557 (134)
Experiment 5						
Left	499 (72)	502 (85)	505 (80)	504 (71)	510 (79)	515 (89)
Right	488 (63)	480 (87)	479 (74)	489 (84)	483 (81)	480 (74)

To investigate the time course of arithmetic-space association in addition and subtraction separately, pairwise *t*-tests were conducted for each delay, although the operation × target side × delay interaction was not significant (see Fig. 2A). The results revealed that when preceded by an addition operation, right-side targets were detected significantly faster than left-side targets for 150 ms delay condition, *t*_25_ = 4.666, *p* < 0.001, and this priming effect extended to 300 ms, *t*_25_ = 4.135, *p* < 0.001. For 500 ms delay, the response to right targets was still faster than to left-side targets, but the difference was not significant, *t*_25_ = 1.711, *p* > 0.05. In contrast, when preceded by subtraction problems, targets on the left side were detected faster than right-side targets, but this effect was only significant at a 300 ms delay, *t*_25_ = −2.678, *p* < 0.05.

Given that the target detection task stimuli were triggered by oral responses from the mental arithmetic task, it is possible that there are trade-offs between the proposal judgment RTs (i.e., arithmetic) and target detection RTs. We therefore checked the correlation between individual-trial proposal judgment RTs and target detection RTs for Experiment 1 (see Supplementary Materials Fig. S1). Statistical analysis^23^ (with subject as a random effect) revealed a small and non-significant correlation between the two RTs (*r* = 0.105; *p* = 0.729).

Experiment 1 replicated earlier work to test whether mental arithmetic could induce spatial shifts of attention. The results indicated that solving addition problems accelerated responding to right-side targets; solving subtraction problems facilitated responding to left-side targets. However, this finding does not address when exactly the spatial attention shift arises during mental calculation. Indeed, the attention shift may be linked to any element or process involved in mental calculation, including the operands, the operator, or the actual process of transforming the operands into the result. It thus remains to be determined what aspect of mental calculation caused the spatial attention shift. In order to clarify this issue, we conducted the following experiments.

## Experiment 2

### Probing after the second operand, and matching operands in addition and subtraction

Here, we wanted to check whether the arithmetic-space association would occur in the course of calculation. For this purpose, we moved the target detection task prior to making a judgment for the proposal (i.e., after the second operand), and matched operands (both first and second operand) in addition and subtraction.

### Method

#### Participants

The subjects were 26 undergraduates (10 males, 24 right-handed) with a mean age of 22.3 years. One of them was excluded from further analysis because of low accuracy (72%). All participants had normal or corrected to normal vision and had not participated in our previous experiment.

#### Stimuli

We used the materials of Knops and Viarouge^8^ to construct the main arithmetic problems used in Experiment 2. The first operand was 32, 48 or 60. The second operand was generated with a fixed proportion of the first operand: 31%, 45%, or 61%. The operands were matched in addition and subtraction. All other aspects of the stimulus set were the same as in Experiment 1 (see Supplementary Materials Table S2).

#### Task and procedure

The task and procedure were identical to that in Experiment 1, except the location of the target detection task.

### Results and Discussion

13% error and extreme trials were excluded from the following analysis. A 2 × 2 × 3 repeated measures analysis of variance (ANOVA) was carried out on mean RTs of the target detection task. The Mean RTs (and SD) as a function of operation, target side and delay are presented in Table 1; see Fig. 2B for mean effects. Target detection times were longer during subtraction, *F* (1, 24) = 8.974, *p* < 0.05, *η* ^2^ = 0.272. The main effect of delay was also significant, *F* (2, 48) = 6.815, *p* < 0.05, *η* ^2^ = 0.221. Multiple comparison analysis showed that targets were detected fastest at 500 ms delay, which was significantly faster than 150 ms delay, *F* (1, 24) = 17.369, *p* < 0.05, *η* ^2^ = 0.420, but the difference between 300 ms and 500 ms was not significant, *F* (1, 24) = 1.317, *p* > 0.05, *η* ^2^ = 0.052. Importantly, we found a significant interaction between operation and target side, *F* (1, 24) = 14.464, *p* < 0.05, *η* ^2^ = 0.376. Simple effect analysis indicated that targets on the right side were detected faster when solving addition problems, *F* (1, 24) = 4.806, *p* < 0.05, *η* ^2^ = 0.167, while targets on the left side were detected faster during subtraction, *F* (1, 24) = 8.024, *p* < 0.05, *η* ^2^ = 0.251.

The operation × target side × delay interaction was significant, *F* (2, 48) = 7.149, *p* < 0.05, *η* ^2^ = 0.230. We again calculated pairwise *t*-tests to reveal the time course of arithmetic-space association for each operation. As can be seen in Fig. 2B, for addition the response to right-side targets was faster than left-side targets; this difference was found at both 300 ms (*t*_24_ = 2.739, *p* < 0.05) and 500 ms delays (*t*_24_ = 2.567, *p* < 0.05). The arithmetic-space association induced by subtraction was marginally significant in 300 ms delay condition, *t*_24_ = −1.781, *p* = 0.088, but strong in the 500 ms delay condition, *t*_24_ = −4.075, *p* < 0.000.

As predicted, when the target detection task appeared after the second operand, the arithmetic-space association effect arose dynamically. There was no effect at a 150 ms delay. However, an effect appeared at 300 ms delay, and lasted through to the 500 ms delay. This suggests that the arithmetic-space association could occur before the arithmetic operation completely finished.

## Experiment 3

### Probing after the second operand, and matching the second operand and proposal in addition and subtraction

In Experiment 2, an arithmetic-space association effect was observed after the second operand was presented. However, it could be argued that this effect is due to number processing of the second operand or the result (rather than the arithmetic operation), because at that time participants might have generated an answer, and the proposals in addition were always larger than those in subtraction. For this reason, in Experiment 3 we manipulated the first operand to keep the second operand and the proposals the same for addition and subtraction (see Supplementary Materials Table S3). All other experimental details remained unchanged relative to Experiment 2.

### Method

#### Participants

27 undergraduates (12 males, 27 right-handed) with a mean age of 21.6 years participated in Experiment 3. One of them was excluded from further analysis because of low accuracy (79%). All remaining participants had normal or corrected to normal vision and had not participated in the previous experiments.

### Results and Discussion

Error and extreme trials (12.5%) were discarded. The 2 × 2 × 3 repeated measures analysis of variance (ANOVA) revealed that there was a significant difference between addition and subtraction, *F* (1, 25) = 23.291, *p* < 0.05, *η* ^2^ = 0.482, and the main effect of delay was also significant, *F* (2, 50) = 11.518, *p* < 0.05, *η* ^2^ = 0.315. The mean RTs (and SD) as a function of operation, target side and delay are presented in Table 1; see Fig. 2C for mean effects. Multiple comparison analysis showed that the target detection time was shortest at 300 ms delay, which was significantly faster than at 150 ms, *F* (1, 25) = 7.637, *p* < 0.05, *η* ^2^ = 0.234, but the difference between 300 ms and 500 ms condition was not significant, *F* (1, 25) = 1.852, *p* > 0.05, *η* ^2^ = 0.069. Crucially, the interaction between operation and target side was again significant, *F* (1, 25) = 19.619, *p* < 0.05, *η* ^2^ = 0.440. Follow-up analysis indicated that addition facilitated the detection of right-side targets, *F* (1, 25) = 19.433, *p* < 0.05, *η* ^2^ = 0.437, while subtraction accelerated the response to left-side targets, *F* (1, 25) = 3.586, *p* > 0.05, *η* ^2^ = 0.125.

Although the three-way interaction was not significant (*p* > 0.05), pairwise *t*-tests were again conducted to reveal the time course of the effects (see Fig. 2C). For addition, the arithmetic-space association appeared at 150 ms delay (*t*_25_ = 3.703, *p* < 0.05), and lasted to 300 ms (*t*_25_ = 2.893, *p* < 0.05) and 500 ms (*t*_25_ = 2.675, *p* < 0.05). For subtraction, the effect was first observed at 300 ms delay, *t*_25_ = −2.491, *p* < 0.05. At 500 ms delay, participants’ RTs for detecting left-side targets were faster than for right-side targets, but the difference was not significant, *t*_25_ = −1.421, *p* > 0.05.

Consistent with Experiment 2, the results of Experiment 3 confirmed that arithmetic-space associations can occur before the proposal. Importantly, the effect was due to the arithmetic operation, rather than the numbers themselves.

## Experiment 4

### Probing before the second operand, and matching operands in addition and subtraction

To further probe the origin of the arithmetic-space interaction, we next moved the target detection task to right before the second operand. We predicted that the arithmetic-space association could also occur even before the second operand was presented. Stimuli were the same as those in Experiment 2. Otherwise, the task and trial sequence were identical to other experiments.

### Method

#### Participants

27 undergraduates (9 males, 27 right-handed) with a mean age of 21.4 years participated in Experiment 5. Three of them were excluded from further analysis because of low accuracy (about 75%). All remaining participants had normal or corrected to normal vision, and they had not participated in the previous experiments.

### Results and Discussion

Again, we first discarded error and extreme trials (15.5%), then conducted a 2 × 2 × 3 repeated measures analysis of variance (ANOVA) on mean RTs of the target detection task (see Table 1 and Fig. 2D). The main effect of delay was significant, *F* (2, 46) = 3.951, *p* < 0.05, *η* ^2^ = 0.147. Multiple comparison analysis showed that the target detection time was shortest at 500 ms delay, which was significantly faster than 150 ms delay, *F* (1, 23) = 6.092, *p* < 0.05, *η* ^2^ = 0.209, but the difference between 300 ms and 500 ms was not significant, *F* (1, 23) = 0.177, *p* > 0.05, *η* ^2^ = 0.008. The interaction between operation and target side was significant, *F* (1, 23) = 44.633, *p* < 0.05, *η* ^2^ = 0.660. Simple effect analysis indicated that for addition, targets on the right side were detected faster than target on the left side, *F* (1, 23) = 17.825, *p* < 0.01, *η* ^2^ = 0.437; but targets on the left side were detected faster than that on the right side in subtraction, *F* (1, 23) = 7.466, *p* < 0.05, *η* ^2^ = 0.245. Other main effects or interactions were not found.

Although there was no significant operation × target side × delay interaction, we again conducted follow-up pairwise *t*-test to study the time course of the effects (see Fig. 2D). This showed that the arithmetic-space association was only found in addition, which was significant at 300 ms delay condition, *t*_23_ = 4.916, *p* < 0.01, and extended to 500 ms, *t*_23_ = 2.692, *p* < 0.05. The effect was absent in subtraction (all *p* > 0.05). Interestingly, this demonstrates that spatial attention can shift if only an operator and an operand are shown.

## Experiment 5

### Probing before the first operand, and matching the operands in addition and arithmetic

Finally, we investigated whether the mere processing of an operator could induce spatial biases. For this purpose, target detection preceded the first operand (so only the operator was shown at that time; see Fig. 1B). Stimuli were the same as those in Experiment 2. Otherwise, the task and trial sequence were identical to other experiments.

### Method

#### Participants

25 undergraduates (9 males, 25 right-handed) with a mean age of 22 years participated in Experiment 5. One of them was excluded from further analysis because of low accuracy (79%). All remaining participants had normal or corrected to normal vision. They had not participated in the previous experiments.

### Results and Discussion

11.3% error and extreme trials were discarded. In the subsequent analysis, only the main effect of target side was significant *F* (1, 23) = 52.362, *p* < 0.05, *η* ^2^ = 0.695, showing that right-side targets were detected faster than left-side targets. Other main effects or interactions were not found (see Table 1 and Fig. 2E). Hence, mere presentation of an operator did not induce any spatial attention shift. This implies that arithmetic-space association is initiated after the operator and at least one operand are presented.

## General Discussion

We demonstrated that there is an intimate link between space and mental arithmetic. In particular, during and directly after mental arithmetic, subjects’ spatial attention was directed leftward for subtraction, and rightward for addition. Of course, other studies had suggested this before. However, our study stands out in at least four important respects. First, we systematically moved the target detection task relative to the proposal, operands and operator. Second, we manipulated three variable delay times between the two tasks. Third, we rigorously and systematically controlled the magnitudes of operands and proposals. Indeed, the magnitudes of proposals were matched in addition and subtraction (Experiment 1); or the magnitudes of operands (Experiment 2, 4); or the magnitudes of the second operand and proposals (Experiment 3). Fourth, we explicitly demonstrated that in our paradigm, the mere processing of an operator did not induce a leftward or rightward shift (see Experiment 5). For these reasons, we were the first to really pinpoint the arithmetic-space interaction in mental arithmetic, rather than in the numbers or the operator.

In addition to this novelty, our results also confirmed earlier research in several respects. Consistent with the results reported by Mathieu *et al*.^18^, the arithmetic-space association effect arose earlier in addition than subtraction. The spatial biases induced by arithmetic operation were uniformly robust at the 300 ms delay and faded away with delays of 500 ms, consistent with the temporal course of number-space interactions found in Fischer *et al*.^10^. Also, rightward responses were consistently faster than leftward ones, probably due to the fact that most of our participants are right-handed. Moreover, shifts of attention for subtraction (i.e., leftward) were generally smaller than those for addition. It is not clear why this is so. One reason could be that subtraction is typically more difficult than addition (consistent in the five experiments; the results of pairwise t-test on mean RTs of the arithmetic task with operation as within-subject factor can be seen in Supplementary Materials Table S5). For this reason, participants may have needed more effort or working memory in subtraction than in addition calculations. This extra load might have diminished our experimental effect size in the subtraction condition.

Arithmetic-space interactions have typically been interpreted as movement along a mental number line^2, 9, 17, 24^, with the direction of movement dependent on the operation. Alternatively, our earlier neuro-computational model^6^ suggests that a neural network for (shifting) spatial attention is the basis of mental arithmetic. This spatial neural network would be recycled during mental arithmetic. Interestingly, just one number and an operand were sufficient to generate an attention shift. In the neuro-computational model, this makes sense because if an operand and one of the numbers are specified, then this number can be “compared” with the prior activation distribution in the other number representation, leading to activation in the arithmetic output, and thus to activation at motor level. Another but related explanation for the finding that an operator and one operand are sufficient to generate an attention shift is that such shifts would occur only when a numerical reference (deictic center) is available. In particular, when a participant has been presented with an operator and an operand, a representational space with reference (i.e., the first operand), and thus left and right options would be activated. These possibilities remain to be disentangled in future work.

Although the arithmetic-space association was quite robust in our studies, several issues need to be addressed in future work. First, operands and results used in our study were mostly two-digit numbers. Two-digit arithmetic prevents subjects from using procedural knowledge to solve simple arithmetic problems and retrieve the results from long-term memory without counting^16, 25^. Future research should investigate whether similar results hold for single-digit arithmetic and for multi-digit arithmetic more generally. Second, the spatial organization of number magnitude is very sensitive to cultural factors^26, 27^. Whether the spatial shifts of attention in mental arithmetic relate to cultural factors remains an open question. Third, eye movements should be measured during mental arithmetic in future research. In this way, one can investigate whether the spatial attention shifts occur up to motor level. Fourth, electro-encephalography could provide a more continuous measure of cognitive processing than is possible with behavioral methods. In this way, the locus of attention shifts may be examined with still higher temporal resolution.

Finally, why would the cognitive system implement mental arithmetic on spatial transformation machinery? Theoretical (neural network) studies have shown that recycling is computationally efficient^28–31^. Moreover, much data has been garnered at both neural^7, 32, 33^ and behavioral levels for the recycling hypothesis^34–36^. Mental arithmetic and space may just be one instance of this general phenomenon.

## Electronic supplementary material

Supplementary Materials
