# Supplementary material for: The Time Course of Spatial Attention Shifts in Elementary Arithmetic
Source: Sci Rep. 2017 Apr 19;7:921. doi: 10.1038/s41598-017-01037-3 (PMC5430428; doi:10.1038/s41598-017-01037-3)

Supplementary Materials

The Time Course of Spatial Attention Shifts in Elementary Arithmetic

Dixiu Liu[1, 2, 3], Danni Cai[1, 2, 3], Tom Verguts[4] and Qi Chen[1, 2, 3*]

[1]School of Psychology, South China Normal University, 510631 Guangzhou, China

[2]Center for Studies of Psychological Application, South China Normal University, 510631 Guangzhou, China

[3]Guangdong Key Laboratory of Mental Health and Cognitive Science, South China Normal University, 510631 Guangzhou, China

[4]Department of Experimental Psychology, Ghent University, 9000 Ghent, Belgium

Correspondence: Qi Chen

School of Psychology

South China Normal University

510631 Guangzhou

P.R. China

Email: chen.qi@m.scnu.edu.cn

Table S1

All Arithmetic Problems Presented in Experiment 1 and Their Correct and Deviant Results.

Operands		Proposals				
O1	O2	1/1.4	1/1.2	1/1	1.2/1	1.4/1
		Addition				
14	5	13	16	19	23	27
14	7	15	18	21	25	30
14	11	18	21	25	30	35
28	7	25	29	35	42	49
28	13	29	34	41	49	58
28	21	35	41	49	58	69
56	13	49	58	69	82	98
56	28	59	71	84	100	119
56	42	69	82	98	117	139
		Subtraction				
32	13	13	16	19	23	27
32	11	15	18	21	25	30
32	7	18	21	25	30	35
64	29	25	29	35	42	49
64	23	29	34	41	49	58
64	15	35	41	49	58	69
128	59	49	58	69	82	98
128	44	59	71	84	100	119
128	30	69	82	98	117	139

Table S2

All Arithmetic Problems Presented in Experiment 2 and Their Correct and Deviant Results

Operands		Proposals				
O1	O2	1/1.4	1/1.2	1/1	1.2/1	1.4/1
			Addition			
32	9	29	34	41	49	58
32	14	33	39	46	55	65
32	19	36	43	51	61	72
48	15	45	53	63	75	89
48	21	49	58	69	82	98
48	29	54	65	77	92	109
60	19	56	66	79	94	112
60	29	63	75	89	106	126
60	38	69	82	98	117	139
			Subtraction			
32	9	16	19	23	27	33
32	14	13	15	18	21	25
32	19	9	11	13	15	18
48	15	23	28	33	39	47
48	21	19	23	27	32	38
48	29	13	16	19	23	27
60	19	29	34	41	49	58
60	29	22	26	31	37	44
60	38	16	18	22	26	31

Table S3

All Arithmetic Problems Presented in Experiment 3 and Their Correct and Deviant Results

Operands		Proposals				
O1	O2	1/1.4	1/1.2	1/1	1.2/1	1.4/1
			Addition			
14	5	13	16	19	23	27
14	7	15	18	21	25	30
14	11	18	21	25	30	35
28	7	25	29	35	42	49
28	13	29	34	41	49	58
28	21	35	41	49	58	69
56	13	49	58	69	82	98
56	28	59	71	84	100	119
56	42	69	82	98	117	139
			Subtraction			
24	5	13	16	19	23	27
28	7	15	18	21	25	30
36	11	18	21	25	30	35
42	7	25	29	35	42	49
54	13	29	34	41	49	58
70	21	35	41	49	58	69
82	13	49	58	69	82	98
112	28	59	71	84	100	119
140	42	69	82	98	117	139

Table S4

Mean RT (and SD) of the proposal judgment task as a function of Operation, Target side, and Delay (in ms) in five experiments.

		Addition			Subtraction		
		150	300	500	150	300	500
Experiment 1	Left	865	859	942	1047	999	1035
		(240)	(276)	(350)	(377)	(341)	(328)
	Right	891	878	960	1076	1044	1071
		(291)	(277)	(365)	(359)	(331)	(377)
Experiment 2	Left	725	756	748	799	780	792
		(238)	(296)	(300)	(336)	(289)	(298)
	Right	719	692	744	783	770	797
		(245)	(225)	(301)	(318)	(274)	(337)
Experiment 3	Left	593	599	597	654	601	620
		(92)	(96)	(105)	(107)	(119)	(103)
	Right	613	590	605	649	606	645
		(94)	(92)	(99)	(122)	(112)	(131)
Experiment 4	Left	1051	1066	1077	1092	1119	1076
		(270)	(338)	(296)	(319)	(333)	(299)
	Right	982	1055	1079	1102	1118	1152
		(234)	(338)	(292)	(269)	(295)	(341)
Experiment 5	Left	868	869	901	1002	959	1048
		(197)	(187)	(254)	(310)	(252)	(331)
	Right	834	865	929	1101	1043	1088
		(163)	(207)	(271)	(340)	(262)	(327)

Note: The proposal judgment task was followed by target detection task except Experiment 1.

Table S5

Mean RT (and SD) of the result judgment task as a function of Operation, and the T-test results in five experiments.

Experiment	arithmetic	Descriptive Statistic			T-test	
		N	Mean (ms)	SD (ms)	*t*	*p*
1	Addition	26	961	339		
	Subtraction	26	1135	468	7.556	.000
2	Addition	25	753	286		
	Subtraction	25	820	359	3.393	.002
3	Addition	26	603	92		
	Subtraction	26	642	126	4.02	.000
4	Addition	24	1041	314		
	Subtraction	24	1123	363	4.28	.000
5	Addition	24	958	263		
	Subtraction	24	1137	332	7.01	.000

Figure S1

All individual oral latencies (Judgment RT) against target detection times (Detection RT) in different SOA (150, 300 and 500 ms) for Experiment 1.

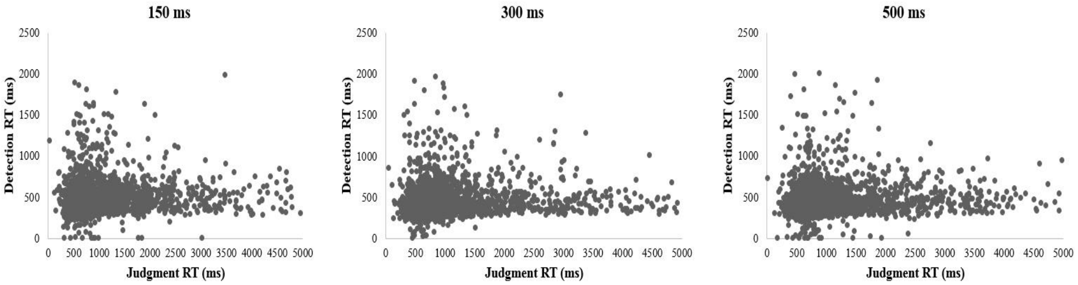

Supplement: Supplementary file 1 — Supplementary Materials [file 41598_2017_1037_MOESM1_ESM.pdf]
